# Effects of mind–body health qigong exercise on depression severity and cognitive function in older adults at a senior care center: a randomized controlled trial

**DOI:** 10.3389/fpubh.2026.1853313

**Published:** 2026-07-07

**Authors:** Longfei Cao, Kai Qi, Xiaoxiao Dong, Chunhui Zhou, Yifan Xu, Aiguo Chen

**Affiliations:** 1 Gdansk University of Physical Education and Sport, Gdansk, Poland; 2 Jiangsu Vocational Institute of Commerce, Nanjing, Jiangsu, China; 3 Nanjing Sport Institute, Nanjing, Jiangsu, China; 4 Soochow University, Suzhou, Jiangsu, China

**Keywords:** cognitive function, depression severity, mind-body health qigong exercise, older adults, senior care center

## Abstract

**Objective:**

This study was conducted to assess the impacts of mind–body Health Qigong exercise performed regularly over a period of 11 weeks upon depression severity and cognitive function among older people.

**Methods:**

A 2 × 2 two-factor structural design was incorporated into this research to carry out a randomized controlled trial. 35 participants underwent random allocation to the intervention arm (*n* = 17) and the control arm (*n* = 18). A regular mind–body Health Qigong exercise intervention carried out over 11 weeks was administered to the experimental group, with a 45-min session performed 3 times each week. The control group was instructed to retain their habitual lifestyle without any intervention. The depression severity and cognitive function of older adults in the experimental and control groups were assessed by means of the 15-item Geriatric Depression Scale and the Chinese version of the Mini-Mental State Examination before and after the exercise intervention.

**Results:**

Compared with baseline, the total scores of C-MMSE in the experimental group were significantly higher at post-intervention (*p* < 0.001), and the scores of its subdomains (orientation and immediate memory) were also significantly elevated (*p* < 0.05); meanwhile, the post-test total scores of GDS-15 in the experimental group was significantly reduced (*p* < 0.01). In contrast, the pretest and posttest total scores of C-MMSE in the control group did not differ notably (*p* > 0.05), but the scores of its subdomain (delayed recall) were significantly decreased (*p* < 0.05); furthermore, the control group exhibited a notable rise in the post-test total scores of GDS-15 (*p* < 0.01).

**Conclusion:**

The results indicated that engaging in mind–body Health Qigong Taiji Stick exercise for 11 weeks yielded statistically significant improvements in cognitive subdomains, including orientation and immediate memory, as well as a statistically significant reduction in depression severity among older adults. The clinical significance of these beneficial effects remains uncertain and should be confirmed by future studies with larger sample sizes.

**Clinical trial registration:**

This study was approved for registration by the Chinese Clinical Trial Registry on April 24, 2024 (ChiCTR2400083424).

## 1 Introduction

Global epidemiological data revealed that 7% of those aged 60 and above were affected by depression (1), and the corresponding rate of mild cognitive impairment ranged from 10 to 20% (2). Clinical studies demonstrated that varying degrees of depressive symptoms were frequently experienced by individuals with mild cognitive impairment (3), while older patients with depression frequently exhibited concomitant cognitive impairment, and higher suicide and mortality rates were observed in this subgroup (4). Moreover, it has also been observed that up to 32% of individuals with MCI developed Alzheimer’s disease over 5 years, and this risk increased significantly over time (2). Therefore, early combined screening for cognitive function and depressive severity among older adults, followed by timely targeted therapies, can effectively enhance their healthy status and life quality, while substantially alleviating the economic and manpower burdens of long-term care.

The common therapies for depression and cognitive impairment for older adults include pharmacological and non-pharmacological therapies, among which cognitive behavioral and physical exercise interventions are the primary non-pharmacological approaches (5, 6). Although pharmacological therapy can rapidly alleviate depressive symptoms, long-term administration is frequently accompanied by a wide range of adverse events, including sleep disturbances, sexual dysfunction, bleeding, and an elevated risk of fractures (7–9). Similarly, pharmacological interventions for cognitive impairment are also linked to various adverse events, such as dizziness, nausea, and diarrhea, showing a dose-dependent increase in their incidence rate (6). Given the potential side effects of pharmacotherapy, older adults are more inclined to choose non-pharmacological interventions to relieve depressive symptoms and improve cognitive function. Compared with cognitive behavioral interventions, exercise interventions have greater advantages in terms of implementation convenience, accessibility, and the feasibility of simultaneous group delivery. Thus, exercise interventions deserve priority consideration in non-pharmacological intervention studies targeting depressive symptoms and cognitive function among older adults.

Multiple studies have verified that performing Chinese traditional mind–body exercises, including Yijinjing, Baduanjin, and Tai Chi, can effectively alleviate depressive symptoms (10–12) and enhance cognitive function among older adults (13–15). Taiji Stick utilized in this study is also a form of Chinese traditional mind–body exercises, and simultaneously it has been officially included in the compiled and promoted Health Qigong program by China’s General Sports Administration, alongside exercises such as Baduanjin and Yijinjing of particular importance, within the eleven sets of Health Qigong advocated by China’s General Sports Administration, Taiji Stick uniquely employs a wooden implement during practice (16). Chinese traditional mind–body exercises are generally consistent in exercise characteristics (slow movements, low intensity) and exercise philosophies (internal and external cultivation, body–mind unity) (17–19), which has established a firm theoretical and empirical basis for the present research.

Based on the above research background, this study hypothesizes that practicing mind–body Health Qigong may markedly enhance cognitive function and decrease depression severity among older adults. To test this hypothesis, a randomized controlled trial was performed in the present study. As the first study to implement a Taiji Stick intervention among older adults, this research examined its impacts on cognitive function and depression severity. The research gap concerning the impacts of interventions of Health Qigong with implements on cognitive function and depression severity among older adults was addressed by the present study, highlighting its pioneering significance and research value.

## 2 Methods

### 2.1 Study design and registration

This study employed a mixed experimental design with a 2 × 2 two-factor structure, in which group was the between-subjects factor and time was the within-subjects factor. The between-subjects factor comprised two conditions: intervention and control, whereas the within-subjects factor included pre-intervention and post-intervention time points. This experiment was structured into three steps: pre-test, 11 weeks of Health Qigong Taiji Stick practice, and post-test. This research was conducted from February to May 2025 at a residential care home named Zhongshan Meiyuan, located in Nanjing, Jiangsu Province, China. Ethical clearance for this study was granted by Nanjing Sport Institute’s Human Research Ethics Committee (Approval No. RT-2025-04). All procedures were performed in strict compliance with the guidelines of the Declaration of Helsinki. Each participant signed a written informed consent form. The Chinese Clinical Trial Registry approved the registration of this research (Registration No. ChiCTR2400083424) prior to participant recruitment.

### 2.2 Participants

In this study, G*Power 3.1 software was used to calculate the required sample size, with a statistical power of 0.80, an effect size of 0.25, and a significance level of 0.05 for ANOVA of repeated-measures, which showed that at least 34 older adults would be necessary. (1) Inclusion Criteria: ① Age ≥60 years, with no restriction on gender; ② Intact limbs; ③ Having an educational background no lower than junior high school; ④ C-MMSE total score ranging from 19 to 28 (20, 21); ⑤ No previous experience with Taiji Stick practice; ⑥ Voluntary participation in this study and signed informed consent. (2) Exclusion Criteria: ① Severe mental or physical illness; ② Severe visual impairment; ③ Unable to stand and walk without assistance; ④ Lower extremity fracture within the past 6 months. (3) Elimination Criteria: ① Withdrawal from the study midway for any reason; ② Three consecutive absences or a cumulative number of six absences at any point in the course of the intervention.

**Figure 1:**
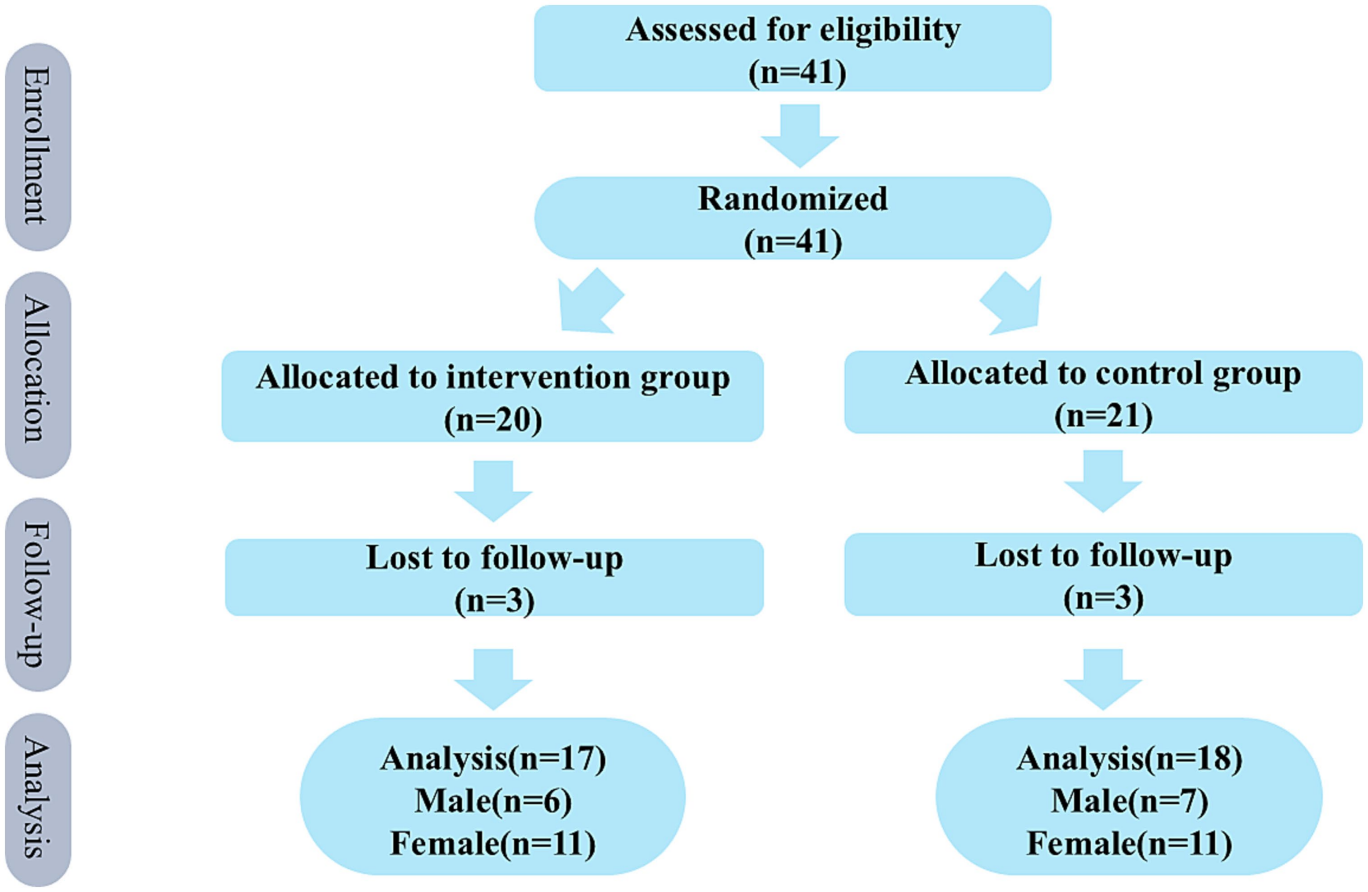
Participant flow chart.

This research recruited 41 older adults who satisfied the eligibility requirements from a senior care center. Prior to randomization, we stratified participants by gender into a male group (15 participants) and a female group (26 participants). Two opaque boxes labeled Box A and Box B were prepared, each with a circular hand-accessible opening on the top. Box A contained 15 uniquely numbered cards ranging from 1 to 15, and Box B contained 26 uniquely numbered cards from 1 to 26. Each male participant drew one card randomly from Box A, and each female participant drew a card from Box B; the number on the drawn card was used as each participant’s unique identification code. Subsequently, two identical sealed opaque envelopes were prepared by a senior care center staff not involved in the research. Each envelope enclosed a distinct grouping rule: one indicated that participants with odd numbers were assigned to the intervention group and those with even numbers to the control group, while the other set the opposite rule whereby even numbers were allocated to the intervention group and odd numbers to the control group. Finally, a Taiji Stick instructor who did not know any of the participants randomly selected one envelope, opened it on-site, and participant grouping was finalized strictly according to the rule inside the envelope. Twenty participants (seven males, thirteen females) were allocated to the intervention group, and twenty-one participants (eight males, thirteen females) were assigned to the control group. During the intervention, 3 participants dropped out from each group. Ultimately, 35 older adults completed the study and entered the data analysis, comprising 17 (six males, eleven females) for the intervention and 18 (seven males, eleven females) for the control (Figure 1). An attrition rate of 14.63% was observed in the present research, which was below the widely accepted 20% threshold for attrition in clinical and exercise science research (22). The compliance rate in the intervention group was 95.72%, with a mean of 31.59 intervention sessions completed.

### 2.3 Intervention program

**Figure 2:**
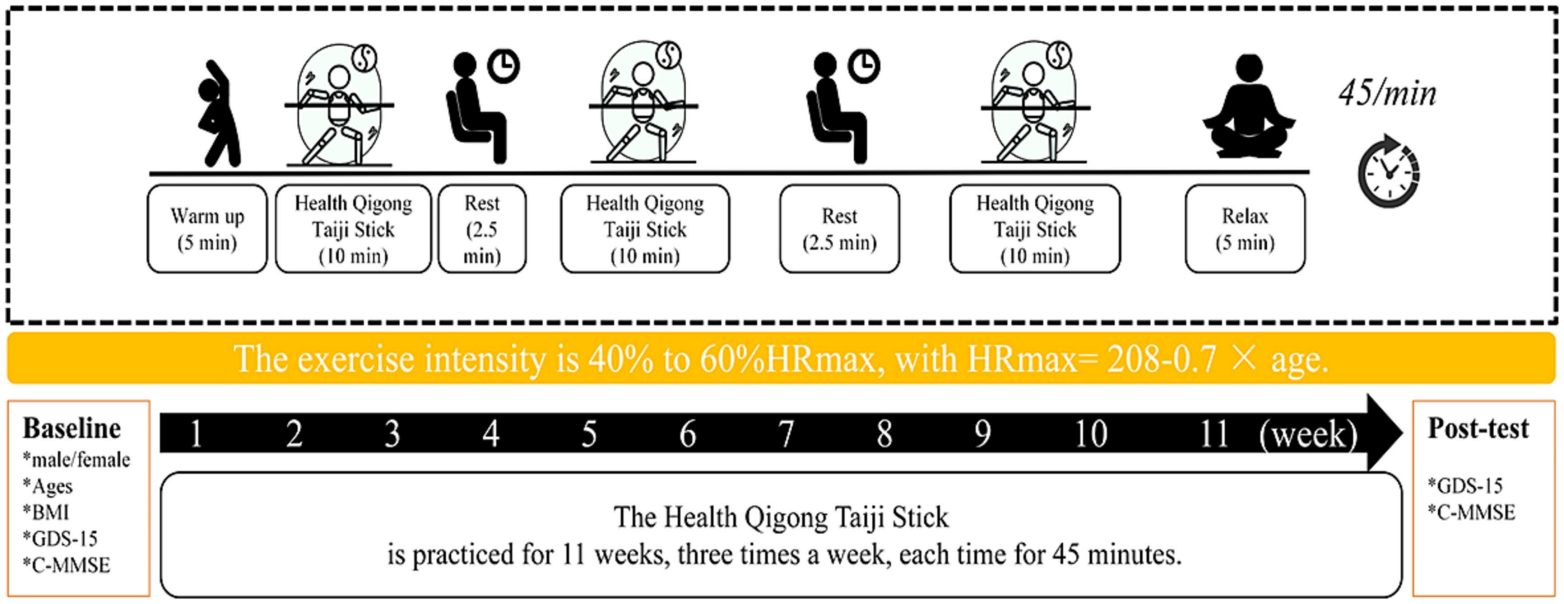
Each session intervention procedure.

Health Qigong Taiji Stick exercise was performed by the intervention group for 11 weeks, Taiji Sticks are crafted from wood and weigh approximately 0.42 kg per stick. Three training sessions were conducted weekly on Monday, Wednesday, and Friday, with 45 min each and commencing at 9:00 a.m. A single 45-min training session consisted of a specific sequence of activities: it commenced with a 5-min preparatory practice, followed by three 10-min rounds of Health Qigong Taiji Stick exercise, each round of exercise was separated by a 2.5-min rest interval, and the session concluded with a 5-min cool-down practice (Figure 2). Each session maintained an exercise intensity of 40–60% of HRmax, where HRmax was derived from the formula 208–0.7 × age (23). Health Qigong Taiji Stick exercise consists of the following 8 forms: 1. Shao Gong Yao Lu, 2. Qing Zhou Huan Xing, 3. Feng Bai He Ye, 4. Chuan Fu Bei Qian, 5. Shen Zhen Ding Hai, 6. Jin Long Jiao Wei, 7. Tan Hai Xun Bao, 8. Qi Gui Dan Tian. Table 1 presents details regarding the general and weekly instruction schedules for Health Qigong Taiji Stick exercise.

**Table 1:** Health Qigong Taiji Stick practicing plan.

Week	Weekly exercise arrangement		
	Monday program wednesday program friday program		
1	Left posture of 1st form	Right posture of 1st form	Full posture of 1st form
2	Left posture of 2nd form	Right posture of 2nd form	Full posture of 2nd form
3	Left posture of 3rd form	Right posture of 3rd form	Full posture of 3rd form
4	Left posture of 4th form	Right posture of 4th form	Full posture of 4th form
5	Left posture of 5th form	Right posture of 5th form	Full posture of 5th form
6	Left posture of 6th form	Right posture of 6th form	Full posture of 6th form
7	Left posture of 7th form	Right posture of 7th form	Full posture of 7th form
8	8th form and closing form	8th form and closing form	8th form and closing form
9	Reinforcement of form 1	Reinforcement of forms 1 to 2	Reinforcement of forms 1 to 3
10	Reinforcement of forms 1 to 4	Reinforcement of forms 1 to 5	Reinforcement of forms 1 to 6
11	Reinforcement of forms 1 to 7	Reinforcement of forms 1 to 8	Reinforcement of forms 1 to 8 and closing form

### 2.4 Outcome assessments

#### 2.4.1 Cognitive function

The C-MMSE was employed to assess cognitive function among older adults. It is an observer-rated scale administered by trained assessors. The validity and reliability of this scale were well established (24, 25). The C-MMSE quantitatively assesses cognitive function based on 5 dimensions: language and copying, recall, attention and calculation, immediate memory, and orientation. Specifically, the orientation dimension is scored from 0 to 10, immediate memory from 0 to 3, attention and calculation from 0 to 5, recall from 0 to 3, and language and copying from 0 to 9. The total score ranges from 0 to 30, with higher scores indicating better cognitive function (26, 27).

#### 2.4.2 Depression severity

Depression severity in older adults was measured using the GDS-15. Its Cronbach’s alpha coefficient is 0.79 and test–retest reliability is 0.73 (28, 29). This scale is a 15-item self-report instrument for depressive symptoms, with each item answered by “yes” or “no” and scored 1 or 0 respectively, and the total score ranging from 0 to 15, where a higher score reflects more severe depressive symptoms. This scale has been widely used in China (30, 31).

### 2.5 Statistical analysis

All statistical analyses were performed with the SPSS 27.0 software. The normal distribution of continuous variables was tested prior to subsequent statistical analyses. Homogeneity test method for baseline data: For the same indicator, if data of two groups were normally distributed, the independent samples *t*-test was used; if data of one group did not conform to a normal distribution, the non-parametric Mann–Whitney *U* test was applied. In addition, to compare the distribution of the categorical variable (gender) between the two groups, a Chi-square test was employed. When the data for each indicator measured before and after the intervention satisfied normality assumptions, ANOVA with repeated measures served to determine intergroup and intragroup differences. In the absence of a significant time × group interaction, main effect analysis was performed; if a significant time × group interaction was observed, simple effect analysis was conducted. To carry out multiple comparisons, post-hoc pairwise analyses were performed using the Bonferroni method. The Greenhouse–Geisser correction was utilized when the sphericity assumption was not satisfied. If the data were not suitable for repeated-measures ANOVA, the non-parametric Wilcoxon signed-rank test was used. Data from repeated-measures ANOVA were presented as Mean ± SD, while data from non-parametric test were presented as Median (Q1, Q3). *p* < 0.05 denoted a significant difference.

## 3 Results

### 3.1 Baseline homogeneity test

At baseline, homogeneity tests were conducted on BMI (kg/m^2^), Age (years), Gender (male/female), total scores and each dimension’s scores of the C-MMSE, and total scores of the GDS-15 between the experimental and control groups. The two groups were comparable at baseline, suggesting homogeneity across the groups (Table 2).

**Table 2:** Baseline characteristics (Mean ± SD)/median (Q1, Q3).

Variables	Intervention group	Control group	χ^2^	T/Z	*p*
*N*	17	18	–	–	–
Gender (male/female)	6/11	7/11	0.048	–	0.826
Age (years)	83.71 ± 2.89	85.22 ± 2.78	–	−1.583	0.123
BMI (kg/m^2^)	23.20 ± 2.99	24.18 ± 3.31	–	−0.922	0.363
C-MMSE (total score)	23.53 ± 2.53	22.89 ± 2.61	–	0.737	0.466
Orientation (score)	7.29 ± 1.86	7.50 ± 1.25	–	−0.386	0.702
Memory (score)	3 (2.5, 3)	3 (3, 3)	–	−0.404	0.687
Calculation (score)	4 (3.5, 5)	4 (3, 5)	–	−0.715	0.475
Recall (score)	2 (1.5, 2)	1 (0.75, 2)	–	−1.383	0.167
Language (score)	8 (7, 8)	8 (7, 8)	–	−0.180	0.858
GDS-15 (total score)	3.24 ± 2.28	3.56 ± 2.01	–	−0.442	0.661

C-MMSE, Chinese version of the Mini-Mental State Examination; GDS-15, Geriatric Depression Scale-15.

### 3.2 Differences in cognitive function and depression severity of the two groups before and after the intervention

#### 3.2.1 C-MMSE total score

The ANOVA involving repeated measures showed that the main effect of time was statistically significant [*F*_(1,33)_ = 31.778, *p* < 0.001, η_p_^2^ = 0.491, 95% CI (0.513, 1.092)], the main effect of group was notable [*F*_(1,33)_ = 4.328, *p* = 0.045 < 0.05, η_p_^2^ = 0.116, 95% CI (0.038, 3.403)], and the time × group interaction was marked [*F*_(1,33)_ = 57.592, *p* < 0.001, η_p_^2^ = 0.636]. Results of the simple effect analysis showed that pre-test C-MMSE total scores were comparable between the experimental group and the control group, with no notable difference detected [*F*_(1,33)_ = 0.543, *p* = 0.466 > 0.05, η_p_^2^ = 0.016, 95%CI (−1.128, 2.409)]; however, the post-test C-MMSE total scores differed significantly between the experimental and control groups [*F*_(1,33)_ = 12.012, *p* = 0.001 < 0.01, η_p_^2^ = 0.267, 95% CI (1.157, 4.445)]. The C-MMSE total scores did not change significantly from pre-test to post-test in the control group [*F*_(1,33)_ = 1.961, *p* = 0.171 > 0.05, η_p_^2^ = 0.056, 95% CI (−0.126, 0.681)]; by comparison, the C-MMSE total scores in the experimental group increased significantly from pre-test to post-test [*F*_(1,33)_ = 85.035, *p* < 0.001, η_p_^2^ = 0.720, 95% CI (1.467, 2.298)]. These results indicated that Health Qigong Taiji Stick training led to a notable improvement in cognitive function among older adults (Table 3).

**Table 3:** Results of ANOVA for repeated measurements (Mean ± SD).

Variables	Intervention group (*n* = 17)		Control group (*n* = 18)	
	Pretest	Posttest	Pretest	Posttest
C-MMSE (total score)	23.53 ± 2.53	25.41 ± 1.97^b^	22.89 ± 2.61	22.61 ± 2.73^d^
GDS-15 (total score)	3.24 ± 2.28	2.18 ± 1.24^b^	3.56 ± 2.01	4.33 ± 2.06^bd^

Posttest compared to pretest within the same group: ^a^(*p* < 0.05), ^b^(*p* < 0.01).

Comparison between groups at the same time point: ^c^(*p* < 0.05), ^d^(*p* < 0.01).

#### 3.2.2 C-MMSE dimension score

According to the results of the Wilcoxon signed-rank test, the experimental group demonstrated notable increases from pre-test scores to post-test scores in two dimensions of the C-MMSE: orientation (Z = −2.150, *p* = 0.032, with an effect size |r| of 0.555) and immediate memory (Z = −2.000, *p* = 0.046, with an effect size |r| of 1.000). In contrast, no significant changes were detected for the remaining dimensions, including attention and calculation (Z = −0.372, *p* = 0.710, with an effect size |*r*| of 0.112), recall (Z = −0.965, *p* = 0.334, with an effect size |*r*| of 0.279), as well as language and copying (Z = −1.660, *p* = 0.097, with an effect size |*r*| of 0.460). Regarding the control group, only the recall dimension of the C-MMSE showed a marked decline from pre-test scores to post-test scores (*Z* = −2.138, *p* = 0.033, with an effect size |*r*| of 0.645). By contrast, the remaining dimensions did not differ markedly: orientation (Z = −0.277, *p* = 0.782, with an effect size |r| of 0.088), immediate memory (Z = −1.000, *p* = 0.317, with an effect size |*r*| of 1.000), attention and calculation (Z = −0.106, *p* = 0.916, with an effect size |*r*| of 0.032), and language and copying (Z = −1.265, *p* = 0.206, with an effect size |*r*| of 0.478). The findings revealed that Health Qigong Taiji Stick practice notably improved orientation and immediate memory in older adults (Table 4).

**Table 4:** Results of wilcoxon signed rank test median (Q1, Q3).

C-MMSE (component score)	Intervention group (*n* = 17)		Control group (*n* = 18)	
	Pretest	Posttest	Pretest	Posttest
Orientation	8 (6, 9)	9 (6.5, 9)^a^	7.5 (7, 8)	8 (7, 8.25)
Memory	3 (2.5, 3)	3 (3, 3)^a^	3 (3, 3)	3 (3, 3)
Calculation	4 (3.5, 5)	4 (4, 5)	4 (3, 5)	4 (3, 5)
Recall	2 (1.5, 2)	2 (1, 3)	1 (0.75, 2)	1 (0, 2)^a^
Language	8 (7, 8)	8 (8, 8.5)	8 (7, 8)	8 (7, 8)

Posttest compared to pretest within the same group: ^a^(*p* < 0.05), ^b^(*p* < 0.01).

#### 3.2.3 GDS-15 total score

The repeated-measures ANOVA demonstrated that the main effect of time was not statistically significant [*F*_(1,33)_ = 0.495, *p* = 0.487 > 0.05, η_p_^2^ = 0.015, 95% CI (−0.266, 0.547)], nor was the main effect of group [*F*_(1,33)_ = 3.935, *p* = 0.056 > 0.05, η_p_^2^ = 0.107, 95% CI (−2.509, 0.032)]; However, a notable time × group interaction was detected [*F*_(1,33)_ = 21.132, *p* < 0.001, η_p_^2^ = 0.390]. Further simple effect analysis revealed no significant difference between the two groups in pre-test GDS-15 scores [*F*_(1,33)_ = 0.195, *p* = 0.661 > 0.05, η_p_^2^ = 0.006, 95% CI (−1.154, 1.795)], but a significant difference emerged in post-test GDS-15 scores [*F*_(1,33)_ = 13.913, *p* = 0.001 < 0.01, η_p_^2^ = 0.297, 95% CI (0.980, 3.333)]; GDS-15 scores demonstrated a marked increase from pre-test to post-test in the control group [*F*_(1,33)_ = 7.803, *p* = 0.009 < 0.01, η_p_^2^ = 0.191, 95% CI (−1.344, −0.211)], whereas the experimental group showed a notable decrease [*F*_(1,33)_ = 13.657, *p* = 0.001 < 0.01, η_p_^2^ = 0.293, 95% CI (0.476, 1.642)]. The above findings suggested that Health Qigong Taiji Stick exercise noticeably reduced depression severity in older adults (Table 3).

### 3.3 Safety assessment

During the entire intervention period, participants did not experience any adverse events related to Health Qigong Taiji Stick training.

## 4 Discussion

As a Chinese traditional mind–body exercise, Health Qigong Taiji Stick features guiding “qi” with the stick, coordinating movements with “qi” circulation, and unifying physical and mental activities. Health Qigong Taiji Stick training emphasizes the waist as the core hub, driving the integrated motions that involve rotating, twisting, bending, and stretching. Findings from this research revealed that older adults who completed a Health Qigong Taiji Stick intervention with forty-five minutes per session, three times each week, lasting 11 weeks, exhibited a marked improvement in cognitive function and a notable reduction in depression severity, thereby validating the initial hypothesis.

Eleven weeks of Health Qigong Taiji Stick intervention led to a substantial enhancement in cognitive function among older adults in the experimental group, which was generally consistent with findings from previous intervention studies employing Baduanjin, Qigong, and Tai Chi. One study has demonstrated that, after participating in Yang-style 8-form Tai Chi practice for 10 weeks (twice weekly, 60 min each), older adults showed a significant enhancement in cognitive performance (32). Another study showed that after engaging in 18 sessions of Qigong exercise over 12 weeks, older adults experienced a marked improvement in cognitive function (33). A trial has shown that older adults with MCI in the community who underwent Baduanjin training for 60 min per session, three times a week over 24 weeks, exhibited a significant improvement in cognitive function (34). Additionally, relevant systematic reviews and meta-analyses have confirmed that older adults engaging in Tai Chi, Baduanjin, or Qigong practice for once to seven times per week, with every time enduring at least twenty minutes, over a period of 6 to 48 weeks, can effectively enhance cognitive function (15, 35).

Based on the generally consistent effects achieved by the aforementioned mind–body exercise interventions, it was necessary to explore the underlying mechanisms by which they enhanced cognitive function among older individuals. Upregulation of BDNF and enhancement of brain neuroplasticity have been proposed as potential mechanisms through which Tai Chi may exert positive impacts against cognitive decline among older adults, according to a systematic review and meta-analysis (36). A study confirmed that Baduanjin exercise exerted positive impact on cognitive performance among older adults. Furthermore, functional near-infrared spectroscopy results revealed a significant elevation in oxyhemoglobin concentration in the left dorsolateral prefrontal cortex (37). A study found that following 12 weeks of Qigong exercise, older adults displayed a significant increase in hippocampal volume and a marked reduction in peripheral interleukin-6 (IL-6) levels. Moreover, a greater improvement in processing speed performance was associated with a larger magnitude of reduction in peripheral IL-6 levels. Meanwhile, the effect of Qigong exercise-induced hippocampal volume augmentation was more prominent in enhancing sustained attention (33). A systematic review pointed out that physical exercise interventions including Tai Chi, Baduanjin, and Qigong could enhance cognitive function and alleviate mild cognitive impairment among older adults via various pathways. These included the activation of signal expression in distinct brain regions, the modulation of connection patterns between these signals, an increase in brain capacity, as well as the regulation of BDNF and inflammatory factors (38).

Multiple meta-analyses have shown that adults who took part in Chinese traditional physical exercises including Yijinjing and Tai Chi for 10 to 24 weeks experienced a marked reduction in depressive symptoms (10, 12). An 8-week Baduanjin intervention (twice weekly, 60 min each session) substantially reduced depressive severity in older adults, as reported by another study (39). Numerous studies explored the mechanisms by which practicing traditional Chinese exercises alleviated depressive symptoms. One study proposed that the regulation of coupling of structure–function in the frontoparietal network and the enhancement of efficiency of the node may underlie the beneficial impacts of Tai Chi practice on subsyndromal depression (40). Another study indicated that demethylation of the BDNF promoter might serve as a potential mechanism through which Tai Chi practice relieved depressive symptoms in community-dwelling older adults (41). Additionally, a research showed that a significant reduction in cortisol levels might mediate the beneficial effects of Qigong practice on depressive symptoms in older adults with chronic physical conditions (42). Chinese traditional physical exercises including Baduanjin and Tai Chi were also categorized as aerobic exercise (43); one study confirmed that aerobic exercise could exert antidepressant effects by increasing the levels of *β*-endorphin, enkephalin, and dynorphin in the human body (44).

On the basis of the above, it can be speculated that the mechanisms underlying improvement in cognitive function and reduction in depression severity induced by Health Qigong Taiji Stick exercise in older adults may be similar to those of the other aforementioned Chinese traditional mind–body exercises.

Physical exercise and mental regulation are the essential attributes of Health Qigong, the mental regulation mechanisms of Health Qigong have been discussed above. It is therefore necessary to further clarify the biomechanical mechanisms through which Health Qigong interventions influence physical function. This will facilitate future large-scale studies on Taiji Stick interventions aimed at exploring the relationships among changes in physical function, cognitive function, and depressive symptoms.

Biomechanical mechanisms shared by Health Qigong in enhancing lower-limb strength among older adults: Baduanjin practice requires practitioners to continuously perform trunk stretching and twisting movements while maintaining postural stability, which helps enhance core strength and trunk stability. In addition, the squatting movements increase the load on the lower limbs by utilizing the practitioner’s own body weight, thereby promoting the activation of lower-limb muscle groups and improving lower-limb strength and stability (45). Wuqinxi practice involves continuous joint flexion and extension, as well as repeated muscle contraction and relaxation, leading to changes in muscle tension and stimulation of mechanoreceptors. Furthermore, Wuqinxi practice can increase motor neuron excitability and positively regulate the function of muscle spindle fibers, thus enhancing lower-limb strength and improving knee joint stability (46). Taiji Stick practice involves various stances, including bow stance, horse stance, empty stance, and resting stance. During practice, frequent transitions between these stances are required, inducing continuous dynamic shifts of the body’s center of gravity in both the mediolateral and vertical directions, thereby increasing loading on lower-limb muscle groups and promoting their activation, ultimately enhancing lower-limb strength (19).

Biomechanical mechanisms shared by Health Qigong in improving gait and balance ability among older adults: Squatting and trunk twisting movements of Ten-Form Health Qigong strengthen core muscles, expand joint range of motion, and boost proprioceptive input in the trunk and lower extremities. During practice, practitioners rotate their bodies bilaterally, stride out and shift the body’s center of gravity to train the waist and knees. Heel raises further activate the gastrocnemius and other calf muscles to build muscle strength while stretching plantar muscles and ligaments. These movements help improve balance ability, enhance gait stability, increase step length and raise walking speed (47). The biomechanical mechanisms by which Baduanjin exercise improves gait and balance ability in older adults with Parkinson’s disease are similar to those of Tai Chi, such as reduced center of gravity sway and center of pressure separation (48). Dynamic stance transitions during Taiji Stick practice can improve gait stability. Additionally, movements involving head rotation, flexion and extension of the cervical spine, shoulder rotation, waist twisting and sinking, as well as flexion and extension of the knee, ankle, and elbow joints, together with stretching of both the upper and lower limbs, help enhance joint flexibility, postural control and balance performance (19).

Although this study achieved some valuable findings, it still had several limitations. First, only 35 older adults completed the study. Although the sample size satisfied the experimental design, its relatively small scale may compromise the robustness of the results. Secondly, since older adults requested full disclosure of the study purpose during recruitment, blinding could not be performed for participants in this research. Thus, a certain level of measurement bias might be introduced in the assessment of the outcomes. In order to minimize measurement bias resulting from assessors’ expectancy effect, assessor blinding was adopted during the baseline and follow-up administration of the scales. The following strategies were adopted: Nursing professionals from a senior care center performed the assessments, none of whom were informed about the study aim or participants’ group allocation. Following their routine procedures, four nursing staff conducted the scale assessments on the participants included in the present research. Thirdly, the long-lasting effects following the conclusion of Health Qigong Taiji Stick intervention were not evaluated in this study. This research initially intended to explore the impacts of Health Qigong Taiji Stick training with regard to cognitive function and depression severity among older adults at the end of the intervention (immediate effects), thus establishing a basis for future research involving long-term follow-up assessments. The majority of investigations involving Tai Chi intervention have concentrated solely on the immediate post-intervention effects; when long-term sustained effects were examined, a follow-up assessment was commonly scheduled at 3 months after the end of the intervention (49, 50). Therefore, for future studies involving Health Qigong Taiji Stick intervention, in addition to assessing the immediate effects of the intervention, we will also carry out a subsequent assessment at the 3-month time point following intervention to examine the persistence of the beneficial effects, thereby more comprehensively demonstrating the practical value of Health Qigong Taiji Stick exercise.

Building upon the present study, future research will collaborate with multiple senior care centers to recruit a larger sample of older adults for a randomized controlled trial. In addition, a health education control group and a jogging control group will be included to ensure that participation frequency, interpersonal interaction, and research attention are kept as consistent as possible across groups. This design will help mitigate the potential influence of attentional, placebo, and Hawthorne effects on intervention outcomes, thereby enabling a more objective evaluation of the effects of the Taiji Stick intervention on cognitive function and depression severity among older adults. Furthermore, to provide a more comprehensive scientific explanation for the benefits of Health Qigong Taiji Stick exercise, future Taiji Stick intervention studies will also explore the mechanisms underlying its effects on improving cognitive function and reducing depression severity in older adults from the perspectives of neuroscience, brain science, and biology. Additionally, in future studies, we will also conduct a comparative study of Taiji Stick exercise and other unarmed Health Qigong (such as Baduanjin, Wuqinxi, and Yijinjing) among older adults. Through systematic comparative analysis, we will clarify the differences and degrees of the effects of Taiji Stick exercise and unarmed Health Qigong exercise on the biomechanical characteristics of human movement, make up for the deficiencies of this study, and further enrich the biomechanical research evidence of Health Qigong exercise.

## 5 Conclusion

The results indicated that engaging in mind–body Health Qigong Taiji Stick exercise for 11 weeks yielded statistically significant improvements in cognitive subdomains, including orientation and immediate memory, as well as a statistically significant reduction in depression severity among older adults. The clinical significance of these beneficial effects remains uncertain and should be confirmed by future studies with larger sample sizes.

## Data Availability

The original contributions presented in the study are included in the article/supplementary material, further inquiries can be directed to the corresponding author/s.
